# P-600. Lyme Surveillance and Prevention at a Community Health Center in Northeastern Pennsylvania

**DOI:** 10.1093/ofid/ofaf695.813

**Published:** 2026-01-11

**Authors:** Xingzuo Wang, Amninder Singh, Elmkdad Mohammed, Mohamed Khorshid, Nathan Cardona, Mary Louise Decker, Yuexiu Wu

**Affiliations:** The Wright Center for community health, scranton, Pennsylvania; The Wright Center for community health, scranton, Pennsylvania; The Wright Center for community health, scranton, Pennsylvania; The Wright Center for community health, scranton, Pennsylvania; The Wright Center for community health, scranton, Pennsylvania; The Wright Center for community health, scranton, Pennsylvania; The Wright Center for community health, scranton, Pennsylvania

## Abstract

**Background:**

Lyme disease is a tick borne disease transmitted through the bite of infected blacklegged ticks. It can cause a Bull's-eye rash at the site of the bite, fever, headache, fatigue, muscle and joint pain. If left untreated, Lyme disease can lead to severe complications such as arthritis, atrioventricular block, neurological problems, and chronic fatigue. Lyme disease is most common in the northeastern, mid-Atlantic, and upper Midwest regions of the United States. Northeastern Pennsylvania is an endemic area for Lyme diseases.

**Figure 1:**
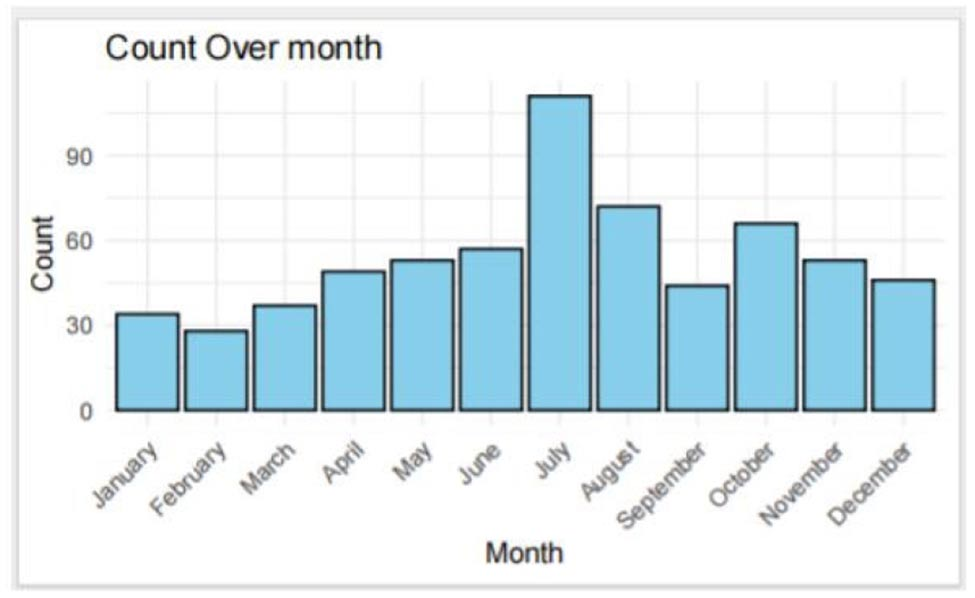
peak incidence of Lyme disease is observed in July. Lyme disease occurs throughout the year. The incidence begins to rise in the spring, peaks in July and then decreases in the fall.

**Figure 2:**
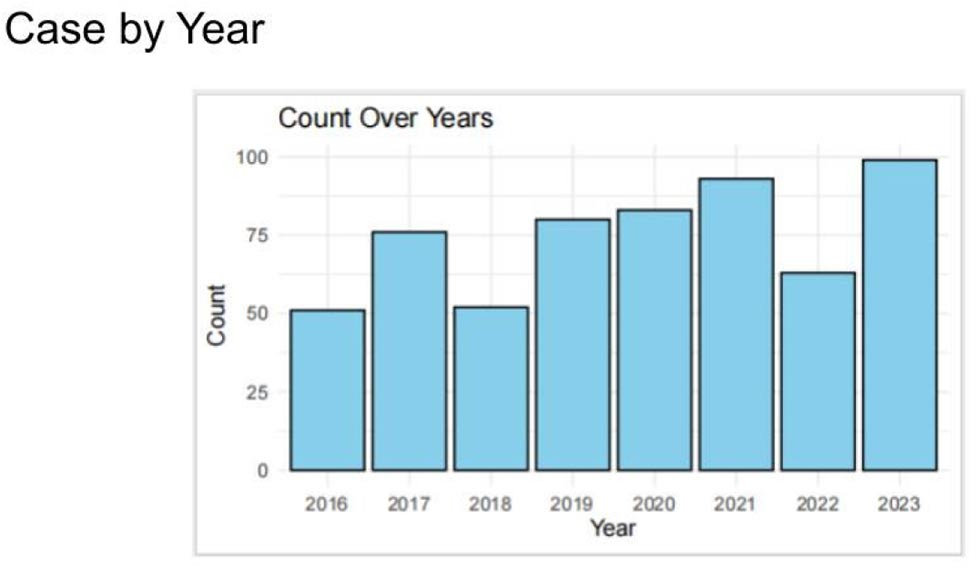
Increased trend of Lyme disease over the years We observed increased trend of Lyme disease in The Wright Center Community Health Clinics from 2016 to 2023.

**Methods:**

Data was collected from The Wright Center community health clinics from 2015 to 2024. ICD-10 code A69.20 was used to extract cases with diagnosis of Lyme disease. RStudio was applied for data analysis. We modified the CDC Lyme disease prevention poster with a QR code for patient education. The poster was uploaded to the TV screens in the clinics.

**Figure 3:**
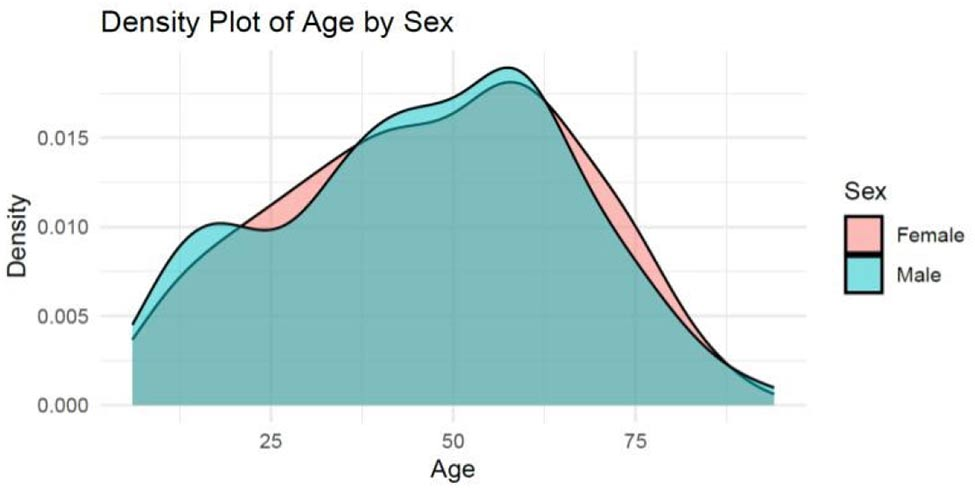
peak distribution of Lyme disease between age 50-60. Incidence of Lyme disease has an age difference with peak distribution between age 50-60 in The Wright Center Community Health Clinics.

**Results:**

Seasonal prevalence of Lyme disease was observed in our study, similar to CDC data. Lyme disease occurs throughout the year. The incidence begins to rise in the spring, peaks in July and then decreases in the fall (Figure 1). We observed an increased trend of Lyme disease over the years in our community (Figure 2). Our data showed the incidence of Lyme disease nearly doubled in 2023 compared with 2016. Incidence of Lyme disease has an age difference with peak distribution between age 50-60 (Figure 3). Peak distribution is different from CDC data which has a bimodal peak distribution in age 5-9 and age 65-69. Lack of the peak distribution in early age is likely due to different patient populations. Majority of the patients in our community health center are above 18 years old, only 20% of the patients are less than 18. Sex difference was also investigated in this study. 55.7% of the cases are female while 44.3% are male. However demographic data showed more female patients than male patients in our clinics. There is no significant sex difference when data is normalized with total female and total male patients respectively.

**Conclusion:**

Lyme disease surveillance is crucial for monitoring disease trends and identifying at-risk populations. Prevention is important to reduce the incidence of Lyme disease and improve community health in endemic areas.

**Disclosures:**

All Authors: No reported disclosures

